# Voltage sensitive currents and information processing by single neurons

**DOI:** 10.1186/1471-2202-16-S1-P158

**Published:** 2015-12-18

**Authors:** Eduard Kuriscak, Zdenek Wunsch, Petr Marsalek

**Affiliations:** 1 Institute of Physiology, First Faculty of Medicine, Charles University in Prague, 128 00, Czech Republic; 2 Inst. of Pathological Physiology, First Faculty of Medicine, Charles University in Prague, 128 53, Czech Republic; 3 Czech Technical University in Prague, Zikova 1903/ 4, 166 36, Czech Republic

## 

This is a computational study using data and models. We study selected neurons in neural circuits, which have different precision of spike timing under reproducible experimental conditions. Voltage sensitive currents govern foremost the spike generation at given times. Yet the effects of voltage sensitive currents are not limited to the axonal hillock, other neuronal structures are also involved. We compare previously described neural computations using models ranked according to level of detail and complexity.

Single neurons propagate neural signal by following steps. The first is the synaptic processing, realized by function of pre- and post- synaptic membrane. Next is signal recoding and encoding and information processing, which is realized by dendrites. As next step follows binary decision spike generation with yes-or-no logical value. This occurs at the axonal hillock. Further signal recoding and encoding is computed by axonal propagation. Finally there is again synaptic processing at the synapse to the next neuron.

In past, some salient features amongst these steps were implemented in biophysically detailed neuronal simulations of anatomically reconstructed neurons. This was achieved via brute-force numerical solution of many parallel instances of cable equation solved by numerical solver of partial differential equations named GENESIS [1]. The experimental observation of voltage dependent ion currents (*active *currents) in dendrites has been a milestone confirming high complexity and high information throughput in single neurons [2].

We use computational description of the pyramidal neuron CA3 and the MSO neuron. The CA3 is anatomical acronym of Cornu Ammonis, 3rd area of hippocampus and the MSO, medial superior olive is a nucleus in the binaural auditory pathway. These model neurons are embodied into three implementations differing by their levels of complexity, as follows.

1. The first level (macroscopic) unit is phenomenological model with black-box components containing elementary arithmetic units connected together, generating spikes as uniform, unitary events [3].

2. The second level (mesoscopic) unit is medium complexity model with delays and voltages described in experiments [4].

3. The third level (microscopic) unit is biophysically realistic detailed model based on the anatomical reconstruction of single neuron [1].

In the three levels we describe necessary time step duration and computational complexity, which has as its practical consequence ration of simulated versus real time on a prototypical contemporary personal computer [5,6]. The implementation details may vary. We use GCC, gcc.gnu.org; www.scholarpedia.org/article/GENESIS[1]; MATLAB (TM), www.mathworks.com; octave, wiki.octave.org[3]; and other software and libraries. In order to abstract the implementation details, we theoretically estimate and recommend the useful range of time step sizes, number of compartments and computational complexity. This enables us to obtain asymptotic and converging figures of information transfer rates and therefore a measure of computational complexity, obtained by the units of three different levels. Finally, we analyze several models in computational neuroscience literature (including our previous works). Based on this analysis, we discuss whether the computational resources were used efficiently.
